# Long-term adoption of plow tillage and green manure improves soil physicochemical properties and optimizes microbial communities under a continuous peanut monoculture system

**DOI:** 10.3389/fmicb.2024.1513528

**Published:** 2025-01-09

**Authors:** Yunfeng Yao, Rongyv Zhu, Xiangdong Li, Guoqing Hu, Yuanjie Dong, Zhaoxin Liu

**Affiliations:** ^1^College of Agriculture, Shandong Agricultural University/National Key Laboratory of Wheat Improvement, Taian, China; ^2^College of Resources and Environment, Shandong Agricultural University, Taian, China

**Keywords:** tillage, green manure, soil nutrient, microbial community composition, continuous cropping constraint

## Abstract

Continuous monocropping of peanuts (*Arachis hypogaea L*.) often results in yield decline and soil degradation. The combination of green manure (GM) with tillage practices has been proposed as a sustainable strategy to maintain high crop productivity and improve soil quality. This study investigates the long-term effects of 8 years of GM application combined with plow tillage on soil microbial communities and physicochemical properties under a peanut monocropping system. Treatments included: (i) no tillage (NT); (ii) plow tillage before the winter fallow period (PT); and (iii) growing ryegrass (*Lolium perenne L*.) during the winter period and applying it as GM before planting next-stubble peanut (PTGM). It was found that both PTGM and PT remarkably decreased the average bulk density (BD), while elevated the mean soil porosity (SP) in 0–30 cm soil layer. Moreover, PTGM significantly increased available potassium (AK), available phosphorus (AP), total nitrogen (TN), and soil organic matter (SOM). Peanut pod yields in the PTGM treatment were 14.1 and 7.2% higher compared to the PT and NT treatments, respectively. Additionally, PTGM could promote shifts in soil bacteria compositions, increasing the abundance of Actinobacteria and Firmicutes while reducing that of Chloroflexi. For fungal abundances, PTGM elevated the abundances of Ascomycota and Basidiomycote. Redundancy analysis demonstrated that SOM, TN, AK, and AP were positively related to dominant flora of fungi and bacteria in PTGM, while negatively related to dominant flora of fungi and bacteria in NT. Overall, tillage practices have the potential to reshape the microbial community during the peanut growing season, primarily due to the influence of SOM, TN, and AP content in shaping microbial diversity and composition. Our study highlights that plow tillage combined with GM application may serve as an effective tillage practice in the future to mitigate continuous cropping obstacles by modulating soil microbial communities, improving soil nutrients and fertility, and enhancing crop productivity.

## 1 Introduction

Peanut represents a vital industrial and oil crop, annually cultivated on approximately 25 million hectares worldwide, producing around 46 million tons (FAO, IFAD, UNICEF, WFP, WHO, 2023; Wan and Zhang, 2019). Due to increasing land scarcity and farmers’ pursuit of higher economic returns, peanuts are often grown continuously in the similar fields with no crop rotation, notably prevalent in the North China Plain (Wang, 2018). However, consecutive monocropping can reduce the quality and yield of peanut due to severe soil-borne diseases, nutrient imbalance, and soil degradation (Larkin et al., 2021; Li et al., 2012). In addition, continuous planting of leguminous crops may alter the soil nitrogen pool and lead to reactive nitrogen losses.

Currently, efforts to alleviate the issues associated with continuous planting primarily involve the use of fungicides and special fertilizers. However, these measures can harm agricultural ecosystems and cause environmental issues (Silva et al., 2019; Tan et al., 2020). Agricultural practices (e.g., intercropping and crop rotation) offer safer and more efficient solutions to reduce continuous planting obstacles (Larkin et al., 2021; Wang et al., 2021). For instance, a maize-peanut intercropping system increased available phosphorus (AP), total nitrogen (TN) and soil organic matter (SOM), optimized soil microbial communities and nutrient compositions, and markedly alleviated the continuous cropping issues in peanuts (Zou et al., 2023). The wheat-peanut rotation sustained soil productivity and quality by increasing microbial biomass and improving soil nitrogen and carbon contents (Zhao et al., 2022). Despite these insights, limited studies have been conducted on the role of plow tillage and green manure (GM) additions in mitigating the continuous planting obstacles of peanuts.

Tillage is a common practice that influences physical/chemical characteristics and soil properties, eventually altering the soil microbial community activity and structure (Sarker et al., 2018; Wang et al., 2022). Prior studies have provided valuable insights into the impacts of diverse tillage practices on soil quality, though with differing outcomes. For instance, some studies have found that no tillage improves SOM content (Paustian et al., 2000), total organic carbon (TOC), particulate organic carbon (POC), (Liu et al., 2021), and microbial biomass (Zuber and Villamil, 2016). Özbolat et al. (2023) demonstrated higher fractions of soil macro-aggregates under no tillage compared with traditional tillage. Although these advantages are well-known, they are often observed in the top 5/10 cm of soils only (Simpson et al., 2004; Zheng et al., 2018). Several studies have suggested that no tillage can suppress microbial activities, owing to oxygen and nutrient limitations, resulting in decreased bacterial abundance and biodiversity (Ranjard and Richaume, 2001). Continuous no tillage may also lead to issues such as surface hardening, reducing air-filled pore space, which hinders peanut pegs from penetrating the soil surface and decreases pod numbers per plant (Bogunovic et al., 2018). There is evidence that scientifically and reasonably managed soil tillage regimes can aggregate stability and soil organic carbon (SOC) by incorporating nutrients and alleviating compaction (Sun et al., 2020; Sae-Tun et al., 2022). Moussa-Machraoui et al. (2010) reported that plow tillage practices can remarkably sustain natural resources and enhance the soil environment. Arvidsson et al. (2014) found that winter wheat yields were, on average, 9.8% lower when using no tillage compared to moldboard plowing. However, the association between microbial community composition and soil chemical properties under continuous peanut monocropping remains unclear.

The application of non-legume GM to leguminous cropland may benefit soil quality, particularly by increasing soil organic matter (SOM), phosphorus (P), and nitrogen (N), while maintaining a healthy ecosystem (Sharma et al., 2017; Jayaraman et al., 2021). Long-term green manure application affected nutrient cycling, and the number of rhizobia and sulfate-reducing bacteria increased, which was conducive to increasing the richness and diversity of microbial community (Gao et al., 2015). Several reports have demonstrated that green manure application can markedly decrease soil bulk density and improve porosity (Gao et al., 2013), improve soil quality and aggregate structure (Jayaraman et al., 2021), enhance microbial biomass and enzyme activities (Gao et al., 2015), and alter microbial community composition and structure (Gao et al., 2018). Extensive research has also been conducted on crop yields with GM application. For example, incorporating GM with 80% of the recommended chemical fertilizer significantly increased early and late rice yields by 11.3 and 6.3%, respectively, in a double-rice cropping system compared with using only chemical fertilizer (Zhang et al., 2023). A 12-year long-term GM (hairy vetch)-potato rotation improved potato yields by 30.4% compared to continuous potato cropping (Wang et al., 2022). Gao et al. (2013) reported that using Chinese milk vetch as winter GM enhanced rice yields by 18.8–28.8% in a long-term rice-rice-GM cropping rotation.

In northern China, peanuts are typically grown from May to October, with the winter months often characterized by bare fallow periods. It is hypothesized that using *Lolium perenne L*. as a GM crop during the winter fallow periods and returning it to the soil at the jointing stage may considerably influence the soil microbial community diversity and composition, improve soil nutrients, and increase peanut yield. In this study, an 8-year field experiment with GM application and two conventional tillage practices (NT, PT) was conducted. Our objectives were: (i) to assess the long-term impacts of combining GM application with plow tillage on soil physicochemical properties, peanut yield and nutrient concentrations; (ii) to evaluate the responses of soil bacterial and fungal community structures to different tillage practices; and (iii) to assess the influence of long-term GM application on the association between microbial community composition and soil chemical properties under continuous peanut monocropping. This study aimed to investigate whether green manure application is superior to conventional tillage practices for sustainable peanut production.

## 2 Materials and methods

### 2.1 Experimental location

Long-term field experiments were carried out at the experimental station of Shandong Agricultural University, located in Tai’an City, Shandong Province, China (36°09′ N, 117°09′ E; 128 m.a.s.l.), belonging to the North China Plain. The experiment was conducted from 2016 to 2023 in a region characterized by a temperate continental monsoon climate and brown loam soils. The average annual precipitation is 631.5 mm and the average annual temperature is 13.7°C. Table 1 presents the initial soil properties of the top 30 cm from 2016, prior to the commencement of the field experiments.

**Table 1:** Soil properties under three different tillage practices.

Year	Treatment	pH	SOM	TN	AP	AK	NH+ 4-N	NO- 3-N
			(g kg^−1^)	(g kg^−1^)	(mg kg^−1^)	(mg kg^−1^)	(mg kg^−1^)	(mg kg^−1^)
2016	Initial	7.17 ± 0.02b	9.6 ± 1.05b	0.87 ± 0.01d	26.4 ± 4.1d	87.92 ± 6.3c	0.96 ± 0.09d	15.63 ± 1.1c
2023	NT	6.09 ± 0.03c	10.2 ± 1.32b	0.94 ± 0.01c	33.9 ± 3.1c	95.4 ± 8.2bc	2.24 ± 1.01c	32.02 ± 1.9b
	PT	7.24 ± 0.05b	14.2 ± 2.09a	1.05 ± 0.01b	49.4 ± 2.6b	101.6 ± 9.3b	3.32 ± 1.95b	40.85 ± 1.3a
	PTGM	8.07 ± 0.04a	20.7 ± 1.86a	1.29 ± 0.01a	62.0 ± 8.7a	138.5 ± 11.2a	3.74 ± 0.86a	46.17 ± 2.8a
	*F*-value	30.16**	25.31**	5.04*	30.17**	104.03**	83.16**	25.52*

Values followed by a different lowercase letter within a column are significantly different at *p* < 0.05. ^*^p < 0.05; ^**^p < 0.01.

### 2.2 Experimental design

Three treatments were organized in a randomized block design with 3 replicates: (i) no tillage during winter period (NT); (ii) plow tillage during winter period (PT); and (iii) growing ryegrass (*Lolium perenne L.*) during winter period and applying as GM prior to planting next-stubble peanut (PTGM). Management details for all treatments are provided in Table 2, while the machinery used is depicted in Supplementary Figure S1. GM was sown annually in November and incorporated into the soil in late May, with no fertilizer applied during the ryegrass-growing period. The peanut variety used was Shanhua 108, sown in early May and harvested in early September. Furrow planting with film mulching, a common local practice, was employed. A compound fertilizers (N: P_2_O_5_: K_2_O = 20:15:10; Shandong AgrUnir Fer SciTech, Feicheng, Shandong Province) was applied at a rate of 750 kg ha^−1^, with all fertilizer incorporated before sowing. Furrow spacing was 180 cm, with six rows per ridge, a row spacing of 30 cm, and a plant spacing of 20 cm. Each treatment had three replications, with plot dimensions of 3.6 × 10 m^2^. Herbicide is applied before film coating, while insecticide is sprayed manually after pests emerge during the later stages.

**Table 2:** The management details of all the treatments.

Treatment	Operation procedure
NT	During the winter fallow period, conventional bare fallow practices were followed, with the soil tilled to a 15-cm depth with a rotary tiller (1GQQN-250GG) to establish a smooth seedbed before planting next-stubble peanut
PT	Following the peanut harvest from the prior growing season, the soil was tilled to a 30-cm depth with a moldboard plow (1LF-435), sun the upturned soil throughout the winter period, and tilled again to a 15-cm depth with a rotary tiller (1GQQN-250GG) to establish a smooth seedbed before planting next-stubble peanut
PTGM	Following the harvest of peanuts from the previous growing season, rotary tillage was conducted to a 15-cm depth with a moldboard plow (1LF-435), and growing ryegrass (*Lolium perenne* L.) during winter period. All the ryegrass plant was cut at a size of 5–8 cm by field straw chopper (1JH-180) and uniformly covered on the topsoil of experimental plots. The experimental plots underwent plowing to a 30-cm depth with a moldboard plow (1LF-435), after which they were tilled two times to a 15-cm depth with a rotary tiller (1GQQN-250GG) to establish a smooth seedbed before planting next-stubble peanut

### 2.3 Soil sampling, soil physicochemical analyses and peanut yield

The sampling design employed a 5-point sampling method, with soil collected at the pod-filling stage from a depth of 0–30 cm using a 20 cm diameter auger in both 2016 and 2023. From each treatment plot, 3 composite random samples were collected, resulting in nine samples per treatment (3 composite samples × 3 plots). The soil samples were sieved through a 2-mm mesh. The aliquot was stored at 4°C for analysis of total nitrogen (TN), available phosphorus (AP), available potassium (AK), pH, ammonium (NH+ 4-N), nitrate (NO- 3-N), and soil organic matter (SOM).

#### 2.3.1 Soil bulk density and soil porosity

Soil BD was measured by the cutting-ring method (Mbuthia et al., 2015). Soil samples were collected from the experimental area between 2021 and 2023. The BD was measured using a stainless steel cutting ring (5 cm in diameter, 5 cm in height, with a total volume of 100 cm^3^) for soil depths of 0–10, 10–20, and 20–30 cm. All soil samples were dried at 105°C until they reached a constant weight to calculate the BD.

$BD\;\left(g\;{\mathrm{cm}}^{-3}\right)=DW/V$

where BD is the soil BD (g cm^−3^); and DW and V represent the dry weight (g) and the volume (100 cm^3^), respectively.

Soil porosity was calculated as a percentage using the following formula (Aziz et al., 2013):

$SP\;\left(\%\right)=\left(1-BD/PD\right)\times 100$

where SP (%), BD (g cm^−3^) and PD (g cm^−3^) are the soil porosity, BD, and particle density (PD was 2.65 g cm^−3^), respectively.

#### 2.3.2 Soil pH, TN, SOM, AK, AP, NH+ 4-N and NO− 3-N concentration

A pH meter was utilized to assess the soil pH using a 1:2.5 soil-to-water solution ratio. Soil organic matter was analyzed through the K_2_Cr_2_O_7_-H_2_SO_4_ oxidation method, while total nitrogen content was determined using the semi-micro Kjeldahl method (Ferreira et al., 2016). Available phosphorus was extracted with NaHCO_3_ and measured via the Mo-Sb colorimetric method using a spectrophotometer (UV2550, Shimadzu, Japan). Available potassium was extracted using NH_4_OAc and quantified by flame photometry. Soil concentrations of NH+ 4-N and NO- 3-N were measured using 25 mL of 1-M KCl solution, which was then filtered through a 0.45 μm membrane filter to remove insoluble particulates, with the concentrations determined using a continuous flow analyzer.

#### 2.3.3 Peanut yields and yield compositions

At the time of peanut harvest, a 7.2 m^2^ quadrat was established in each experimental plot, and the entire peanut crop within this area was excavated to assess the yield. All pods were gathered from the peanut plants, air-dried, weighed, and then adjusted to a standard water content of 8%.

### 2.4 DNA extraction and 16S sequencing

Sampling design used 5-point sampling method and soil was sampled at the pod-filling stage at 0–30 cm depth using an auger (20 cm diameter) in 2016 and 2023. The aliquot was placed on dry ice immediately, transported to the laboratory, and kept at −80°C for later DNA extraction. Total DNA was extracted from 9 specimens acquired from each treatment using an E.Z.N.A.-soil-DNA-Kit (Omega Bio-tek, United States), following the kit’s instructions. The DNA quality and concentrations were assessed using a UV–vis spectrophotometer (Wilmington, DE) after running the samples on a 1% agarose gel. The 338F/806R primer pairs (338F: 5′-ACTCCTACGGGAGGCAGCAG-3′, 806R: 5′-GGACTACHVGGGTWTCTAAT-3′) targeting the V3-V4 hypervariable region of the bacterial 16S rRNA gene, and the ITS1F/ITS2R primer pairs (ITS1F: 5′-CTTGGTCATTTAGAGGAAGTAA-3′, ITS2R: 5-GCTGCGTTCTTCATCGATGC-3′) targeting the ITS1 region of fungal rRNA, were used for amplification. Sequencing of the amplicons was performed by Majorbio BioPharm Technology (Shanghai, China) using the MiSeq PE300 platform (Illumina, United States).

### 2.5 Bioinformatics assessment of raw sequences

The raw sequences were demultiplexed, quality-filtered using FASTQ (v0.20.0), and merged into tags with FLASH (v1.2.7). Tags were clustered into operational taxonomic units (OTUs) using UPARSE (v7.1) at a 97% similarity threshold. Taxonomic classification of each OTU representative sequence was performed using RDP Classifier (v2.2) against the SILVA database (v132) for bacteria and the UNITE database (v8.0) for fungi, with a confidence level set at 0.7.

### 2.6 Statistical analyses

ANOVA was performed using DPS software (v7.05; RuiFeng-Information-Technology, Hangzhou, China). Statistical difference between treatment groups was compared using the least-significant-difference (LSD) test at the *p* < 0.05 level. Principal-coordinate-analysis (PCoA) was carried out according to Bray-Curtis distance matrices. Redundancy-analysis (RDA) was performed to investigate the effect of soil variables on fungal and bacterial communities. PCoA and RDA analyses were carried out on the Majorbio-cloud-platform.^1^ Data visualization was down using SigmaPlot v10.0 (Systat Software, United States).

## 3 Results

### 3.1 Peanut yield

**Figure 1:**
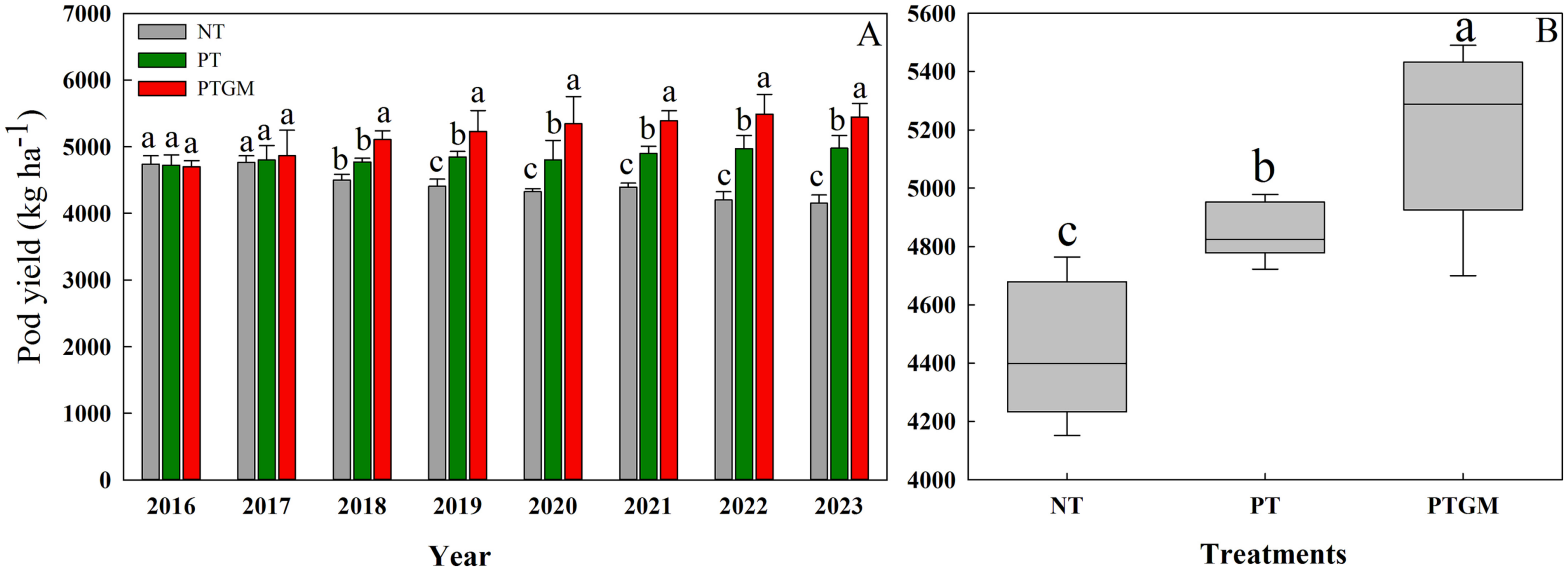
Peanut pod yield and trends in diverse tillage practices from 2016 to 2023 **(A)** and average peanut yield in three tillage practices **(B)**. Significant differences at *p* < 0.05 are denoted by different lowercase letters above each bar. Solid lines in **(B)** indicate mean. Significant differences (*p* < 0.05) among the tillage practices are represented by different lowercase letters.

From 2016 to 2023, the peanut yield in NT treatment decreased from 4,739 kg ha^−1^ in 2016 to 4,351 kg ha^−1^ in 2023. The peanut yield under PT was significantly higher compared to NT, with increases of 6.0%, and the yields exhibited a tendency to remain steady from year to year. In 2016 and 2017, peanut yield showed no significant differences between the three treatments. However, peanut yield with PTGM treatment was significantly higher than those of PT and NT from 2018 to 2023, with an increase from 6.1–20.0% and 7.1–11.3%, respectively (Figure 1A). On an 8-year average, the PTGM increased peanut yield by 7.2 and 14.1% over PT and NT (*p* < 0.05), respectively (Figure 1B).

### 3.2 BD and SP parameters

Notable interaction effects between soil depth and tillage on BD and SP were observed from 2021 to 2023 (Table 3). Bulk density varied significantly (*p* < 0.05) among tillage treatments, with NT demonstrating a remarkably higher BD compared with PT and PTGM. Soil layer depth also significantly affected bulk density (*p* < 0.05), with the 0–10 cm layer showing a markedly lower density compared with the 20–30 cm layer. The most notable reductions in BD were found under the PTGM treatment. Tillage had a substantial impact on SP, with PTGM treatments showing remarkably greater (*p* < 0.05) SP than NT and PT. SP was highest in the 0–10 cm layers and reduced with elevating soil depth to 30 cm.

**Table 3:** Soil BD and soil porosity across diverse tillage practices.

Treatment	2021		2022		2023	
	BD	SP	BD	SP	BD	SP
Tillage (T)	(g m^−3^)	(%)	(g m^−3^)	(%)	(g m^−3^)	(%)
NT	1.57 ± 0.035a	40.67 ± 1.02c	1.50 ± 0.032a	43.31 ± 0.54a	1.49 ± 0.050a	43.79 ± 0.68c
PT	1.46 ± 0.032b	44.61 ± 0.85b	1.39 ± 0.030b	47.4 ± 1.20ab	1.43 ± 0.041b	46.13 ± 0.27b
PTGM	1.41 ± 0.044c	47.22 ± 1.22a	1.35 ± 0.073c	49.08 ± 0.25a	1.32 ± 0.020c	50.06 ± 0.25a
**Soil depth (S)**						
0–10	1.43 ± 0.045c	45.97 ± 0.54a	1.34 ± 0.069c	49.62 ± 0.64a	1.33 ± 0.073c	49.69 ± 0.64a
10–20	1.47 ± 0.030b	44.47 ± 0.80a	1.41 ± 0.045b	46.63 ± 0.63b	1.42 ± 0.044b	46.3 ± 0.28ab
20–30	1.53 ± 0.061a	42.06 ± 0.54b	1.50 ± 0.060a	43.61 ± 0.68c	1.49 ± 0.059a	43.96 ± 0.68b
**ANOVA**						
*F_T_*	25.93**	43.50**	49.60**	35.35**	26.11**	40.18**
*F_S_*	9.30*	15.54**	50.83**	36.12**	21.49**	33.07**
*F_T_* × *F_S_*	0.91	1.16	2.83	0.78	6.08	1.53

Values that are accompanied by different lowercase letters within a column indicate significant differences at *p* < 0.05. *F_T_*, *F_S_*, and *F_T_* × *F_S_* represent the *F*-values for tillage, soil depth, and their interactions in the variance analysis, respectively. For treatment explanations, refer to the text. *p < 0.05; **p < 0.01.

Values are mean ± standard deviation (*n* = 9).

### 3.3 Soil properties

Tillage practices significantly influenced the contents of soil nutrients (SOM, TN, AP, AK, NO− 3-N and NH+ 4-N), and soil pH (Table 1). Specifically, there was an increasing trend in pH under the PTGM treatment from the initial soil pH in 2016 (pH 7.17), whereas pH changed slightly in PT (*p* > 0.05) but decreased slightly in NT. SOM significantly increased in PTGM and PT treatments (*p* < 0.05) but changed slightly in NT. TN ranged from 0.94 to 1.29 g kg^−1^ in 2023, significantly increasing in PTGM and PT treatments (*p* < 0.01). Additionally, AP, AK, NO− 3-N, and NH_4_^+^-N generally showed an increasing trend across the three tillage practices, except for AK in NT. Moreover, SOM, TN, and AP were higher in PTGM and PT treatments than in NT, while AK was significantly higher in PT compared to NT.

### 3.4 Alterations in the microbial community composition under different tillage practices

Tillage practices significantly influenced the *α*-diversity of fungal and bacterial communities (Table 4). The bacterial CE ranged from 3,924 to 4,217, with the maximum value observed in PTGM treatment (*p* < 0.05). The bacterial SI ranged from 6.5291 to 6.8020, with the maximum value in PTGM treatment and the minimum in NT. For fungi, the CE followed the order of PTGM > PT > NT. The fungal SI ranged from 3.7595 to 4.2626, with the maximum value in the PTGM treatment, which was markedly higher than in NT (*p* < 0.05).

**Table 4:** Impact of long-term different tillage practices on the bacterial diversity and quantities.

Treatments	Bacterial		Fungal	
	Chao1	Shannon	Chao1	Shannon
NT	3,924 ± 226c	6.5291 ± 1.35b	703 ± 28c	3.7595 ± 0.39c
PT	4,133 ± 125a	6.7663 ± 1.04ab	741 ± 25b	4.1773 ± 0.20b
PTGM	4,217 ± 167a	6.8020 ± 1.61a	782 ± 13a	4.2626 ± 0.15a
*F*-value	22.57**	0.78*	14.43**	6.35*

Values with distinct lowercase letters in a column indicate an obvious difference at *p* < 0.05. ^*^p < 0.05; **p < 0.01.

### 3.5 Microbial structures and compositions

**Figure 2:**
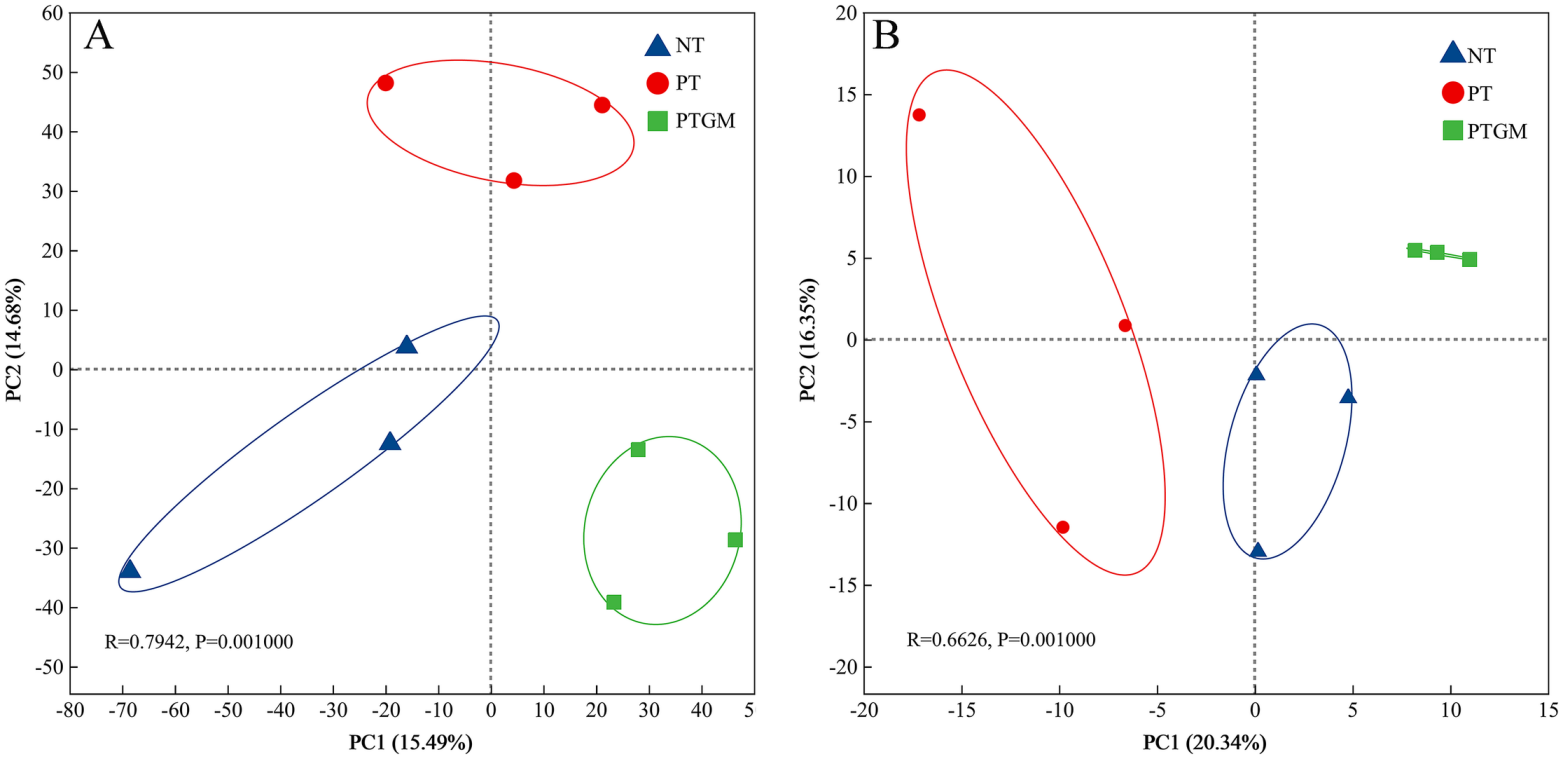
PCoA depicting the clustering of soil bacterial **(A)** and fungal **(B)** communities under diverse tillage practices (OTUs with relative abundance >0.01%).

**Figure 3:**
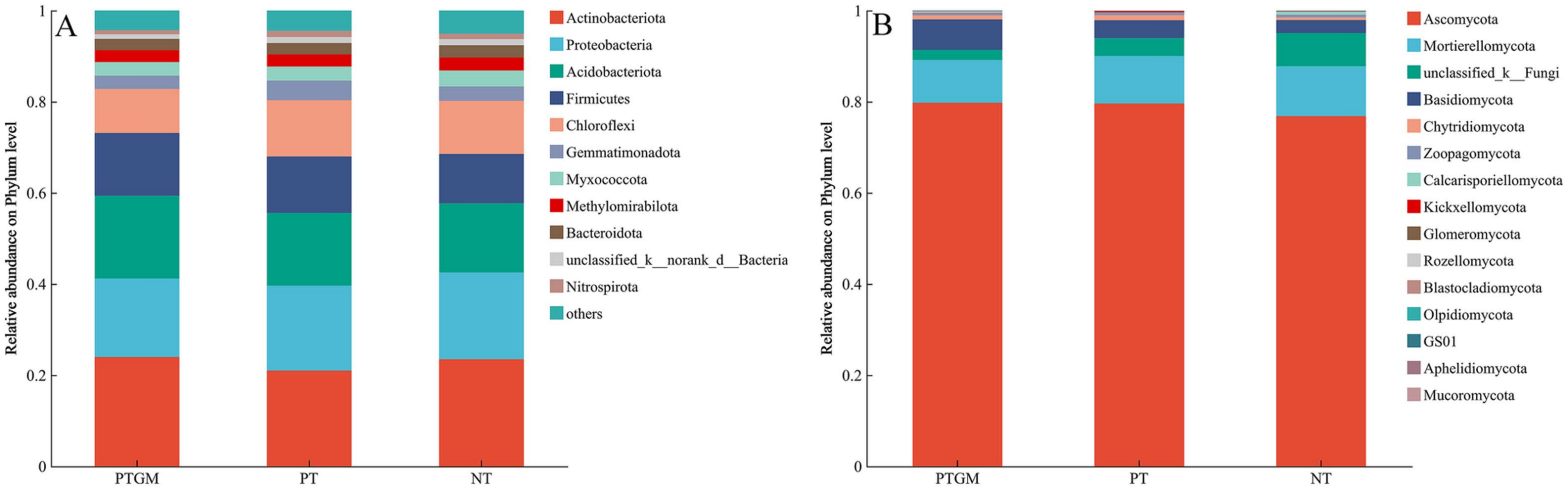
Phylum-level distribution of soil bacterial **(A)** and fungal **(B)** species among diverse tillage practices.

The influence of diverse tillage practices on soil fungal and bacterial community structure was assessed using PCoA (Figure 2). Results indicated distinct clustering of bacterial and fungal communities based on tillage practices, highlighting tillage as a dominant factor shaping microbial community composition. Dominant bacterial phyla across all treatments included Actinobacteriota, Proteobacteria, Acidobacteriota, Firmicutes, and Chloroflexi, collectively accounting for 80.2–82.8% of total OTUs (Figure 3A). Ascomycota predominated among fungal phyla, comprising 76.84–79.77% of total OTUs, followed by Mortierellomycota (9.36–10.92%) and Basidiomycota (2.83–6.68%). Unclassified fungal phyla accounted for 2.24–7.32% of total OTUs (Figure 3B).

The abundance of certain bacterial and fungal phyla varied among tillage practices. Acidobacteriota and Firmicutes showed higher relative abundances in PTGM and PT compared to NT, whereas Chloroflexi showed similar abundances between PT and NT, both higher than PTGM. Other dominant bacterial phyla exhibited no obvious distinctions among the three tillage practices (Figure 3A). For fungi, Ascomycota abundances were relatively similar between PT and PTGM but were higher than in NT. Unclassified-k-Fungi showed significantly higher relative abundance in NT, while Basidiomycota was more abundant in PTGM compared to PT and NT (Figure 3B).

### 3.6 Relationships between soil characteristics and microbial community

**Figure 4:**
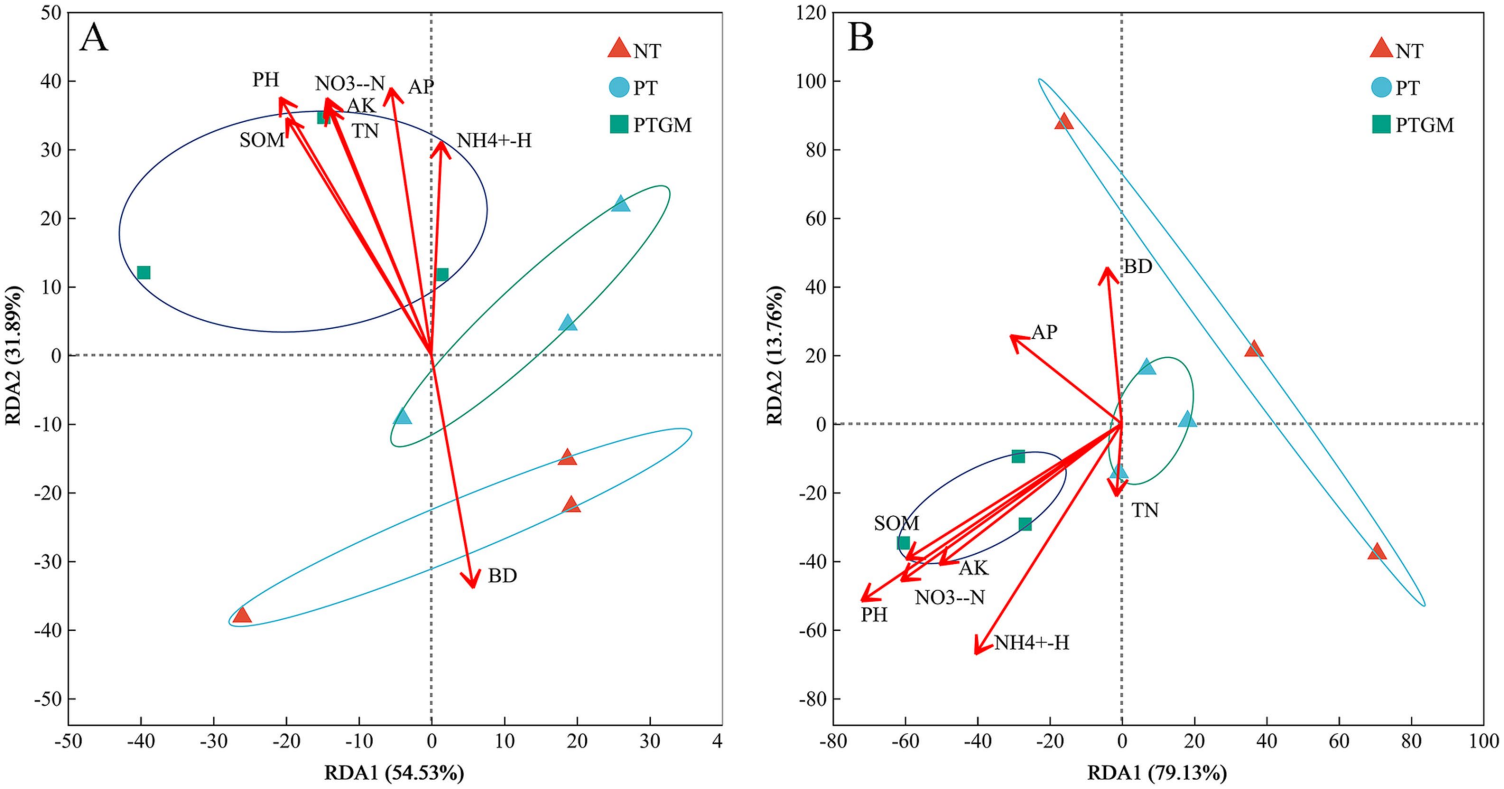
RDA data of bacterial **(A)** and fungal **(B)** community compositions and soil chemical characteristics of diverse tillage practices.

The influence of soil characteristics on the compositions of bacterial and fungal communities was further examined through redundancy analysis, using the selected soil variables and OTU compositions (Figure 4). For bacterial community test, these soil variables accounted for 86.42% of the total variation, with the first two axes explaining 54.53 and 31.89%, respectively. Based on the vectors, bacterial communities in the PTGM treatment were positively associated with SOM, TN, AP, AK, NO− 3-N, NH_4_^+^-N, and pH (Figure 4A). For fungal community test, these soil variables explained 92.89% of the variation, and the first two axes explained 79.13 and 13.76% of the total variation. The fungal communities in PTGM treatment were related to pH, SOM, NO− 3-N, and AK, while it was affected more by AP in PT treatment (Figure 4B).

**Figure 5:**
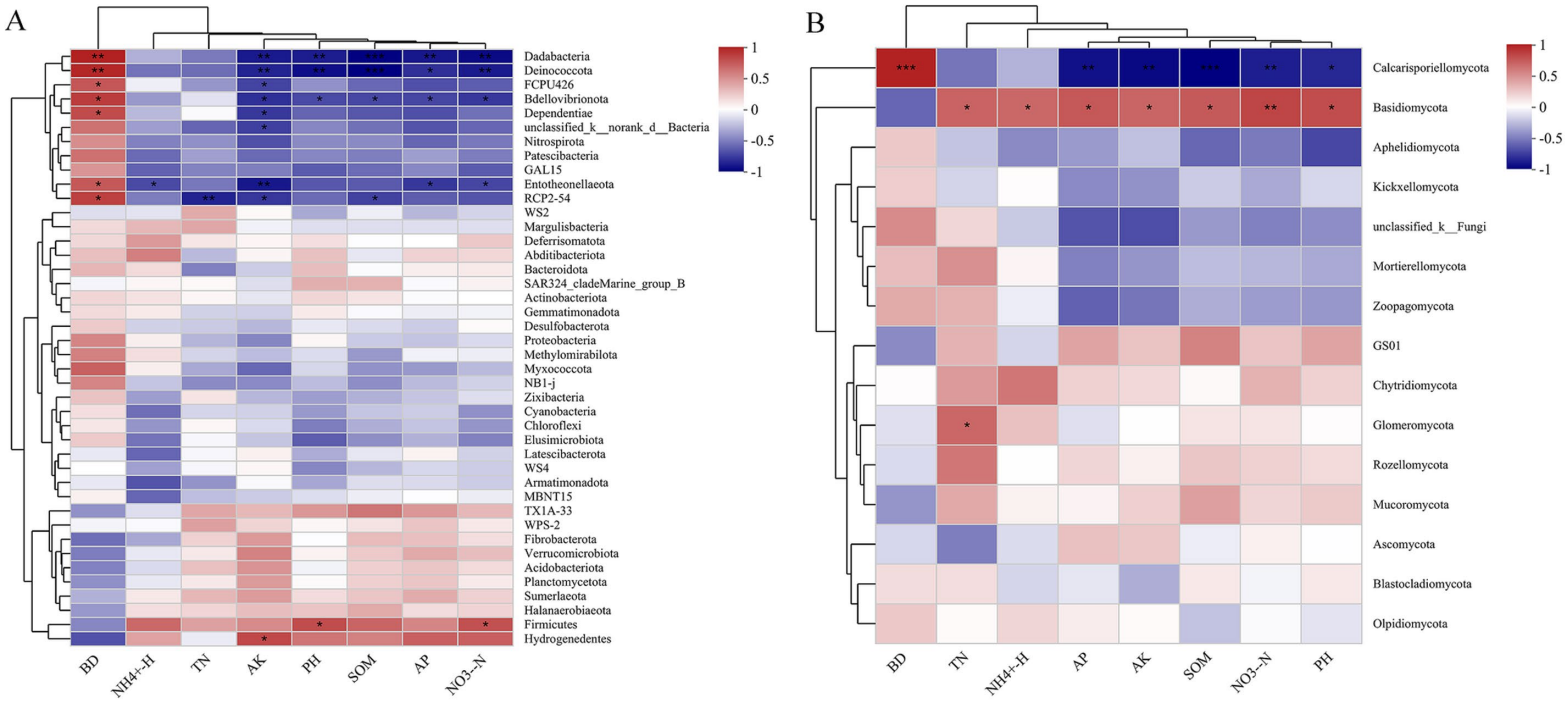
Spearman correlation between genus-level species abundances of bacterial **(A)** and fungal **(B)** species and soil chemical characteristics.

Spearman analysis revealed that soil chemical properties were strongly, yet variably, correlated with the majority of the dominant genera (Figure 5). For the bacterial community genus, the abundances of Dadabacteria and Bdellovibrionota were positively closely related to BD and negatively to the AK, pH, SOM, AP and NO− 3-N. As opposed to Dadabacteria and Bdellovibrionota, the abundances of Firmicutes and Hydrogenedentes were positively related to NO- 3-N and AK, respectively, and negatively associated with BD (Figure 5A). For the fungal community genus, the abundance of Calcarisporiellomycota showed positive correlation with BD and negative correlation with AK, pH, SOM, AP, and NO− 3-N. The Basidiomycota abundance had positive correlation with TN, NH+ 4-N, AK, pH, SOM, AP, NO− 3-N and negatively to the BD. In addition, the abundance of Glomeromycota genera demonstrated a positive correlation with TN (Figure 5B).

## 4 Discussion

### 4.1 Soil physicochemical characteristics

In the practical spring peanut production of the Northern China Plain, fields are typically left bare from the previous peanut harvest until the next sowing season, creating opportune conditions for GM applications (Wan, 2003). Our study demonstrates that GM application remarkably enhances soil physical properties, increases soil fertility, and enriches microbial communities (Tables 3, 4; Figures 2–4).

Soil BD is crucial for assessing soil physical characteristics, particularly for optimal root development (Helliwell et al., 2019). Our findings revealed that the combination of GM application and plow tillage effectively reduced BD, especially within the 0–30 cm soil layers (Table 3), which corresponds to the primary root distribution zone, promoting enhanced root growth and development. Plow tillage is known to decrease soil bulk density by disrupting the argillic horizon layer and alleviating soil compaction. Additionally, the bioturbation caused by GM roots during their growth phase, along with the increases in SOM from residue decomposition, further contributes to reduced soil bulk density (Bassouny and Chen, 2016). Moreover, the average SP in the 0–30 cm layers was found to be 8.2% higher under PTGM and 14.6% higher under PT compared to NT treatments, consistent with previous research (He et al., 2019). These results highlight that the combination of GM application and plow tillage improves soil structure by loosening soil and mitigating compaction, thereby creating a more conducive soil environment for peanut cultivation.

In the Northern China Plain, extensive use of N fertilizer is common to ensure high grain yields, albeit at significant production costs and environmental detriment (Luo et al., 2016). Hence, the impact of GM on soil N content has been a focal point in numerous studies. Our experiment affirms that plow tillage combined with GM effectively enhances available N level in soils across depths of 0–30 cm. Specifically, NH+ 4-N and NO− 3-N concentrations significantly increased under PTGM compared with PT and NT treatments, suggesting a potential reduction in chemical fertilizer requirements to some extent (Couedel et al., 2018). For peanut cultivation, nitrogen uptake encompasses contributions from both fertilizer application and soil sources, including symbiotic N_2_ fixation from the atmosphere, all crucial for growth and production yields (Wang et al., 2016). Therefore, optimizing the balance of soil nitrogen and symbiotic fixation while minimizing fertilizer inputs is imperative for mitigating environmental impacts, though determining the optimal substitution rate of GM for chemical fertilizers requires further investigation. The observed increases in NH+ 4-N and NO− 3-N were related to TN enhancements, consistent with findings in literature (Qiao et al., 2020). GM helps prevent surface runoff but also slows rainwater infiltration during fallow periods, thereby reducing nitrogen leaching. Additionally, vegetation absorbs available nutrients from the soil, while the incorporation of GM enriches soil nitrogen inputs upon decomposition. Other studies have reported similar TN increases following GM applications in maize fields in low fertility arid lands (Yang et al., 2023) and in rice fields on red paddy soils in South China (Zhang et al., 2017).

Furthermore, GM applications remarkably increased SOM, whereas long-term no tillage practices led to a decrease in SOM. Recent research has concentrated on exploring the mechanisms behind these effects. Reports have shown that plow tillage and GM application can enhance soil SOM by 21.4–27.9% compared to reduced or no tillage practices, by promoting enzymatic activities that facilitate nutrient transformations (He et al., 2019; Zhang et al., 2017). In addition, GM applications have been reported to improve nitrogen cycle efficiency and reduce soil nitrogen leaching, thereby enhancing soil quality and functions (Yao et al., 2017). The availability of essential soil nutrients such as phosphorus (P) and potassium (K) was also markedly increased by GM application, particularly in the 0–30 cm soil layers (Table 1). In northern China, where P deficiency can limit crop growth significantly, enhancing available P through agricultural practices is crucial (Hou et al., 2018). Adequate P availability supports crop growth and yield as well as facilitates symbiotic N_2_ fixation in peanuts (Wan, 2003). Additionally, soil pH demonstrated an upward trend under PTGM and PT treatment groups compared with NT. Over the 8-year study period, soil pH decreased from 7.17 in the initial samples (2016) to 6.09 by the final year (2022), reflecting the acidifying effect of continuous monocropping with chemical fertilizer applications, a well-documented phenomenon (Guo et al., 2010).

### 4.2 Peanut yields

Declining crop yields due to continuous monocropping have raised significant concerns (Liu et al., 2008; Nie et al., 2008). In this research, peanut yields remarkably decreased under continuous cropping for 8 years in the NT treatment. However, the application of GM positively influenced subsequent peanut productivity. Initially, there were no significant differences in peanut pod yield among the three tillage practices in the first and second years. However, by the third year after GM application (2018), peanut yields significantly increased in the PTGM treatment. This suggests that PTGM mitigates the negative effects of continuous cropping on yields, with the yield-enhancing effect of GM application becoming more pronounced over time. Similar findings of yield increase with GM application have been reported in rice, potatoes, and maize (Zhang et al., 2023; Wang et al., 2022; Ma et al., 2021).

The beneficial effects of GM on yield are likely attributed to improvements in TN, SOM, AK, and AP contents, along with reductions in BD (Li et al., 2021), which provide essential macro- and micronutrients for crop growth (Özbolat et al., 2023). In addition, plow tillage provides a less restricted soil physical environment for crop root growth than conventional tillage, such as lower soil bulk densities; and this resulted in a better water storage during the peanut season fields, and a higher root length density of peanut, root growth and spatial distribution is vital for water extraction and nutrient uptake (Cai et al., 2014; Mosaddeghi et al., 2009). Because of the improved soil environment and root growth, a greater yield of peanut was achieved with PTGM. Previous studies have also highlighted the yield-promoting effects of long-term GM applications (Zhang et al., 2023). Our findings further underscore the significant and consistent positive impact of GM application on yield improvement across diverse conditions (Raseduzzaman and Jensen, 2017), with PTGM maintaining high yields throughout the experimental period from 2018 to 2023.

### 4.3 Soil bacterial community

Long-term application of GM significantly improved soil physicochemical characteristics as well as affected the compositions and structures of bacterial communities (Zhang et al., 2017; Yang et al., 2023). With years of GM application, soil microbial abundance increased, and PTGM demonstrated higher bacterial richness and Shannon diversity compared to PT and NT (Si et al., 2014). The presence of SOM and mineral nutrients, particularly N, served as essential nutrients and energy sources for microbial growth. Over the 8-year continuous peanut monocropping period, PTGM retained more SOM and mineral nutrients due to reduced nutrient removal by peanut harvest and enhanced input from GM, resulting in higher SOM and TN levels (Table 1) that fostered microbial proliferation in PTGM relative to other treatments. Previous studies have similarly reported that GM applications increase nutrient availability, SOM, and microbial populations (Gao et al., 2018).

The dominant bacterial phyla, such as Proteobacteria, Acidobacteria, Actinobacteria, Chloroflexi, and Firmicutes, are commonly found in soils globally (Maestre et al., 2015). GM application elevated the abundances of Acidobacteria and Firmicutes but reduced those of Chloroflexi. Actinobacteria and Firmicutes exhibited higher abundance in PTGM, indicating a positive impact of GM on these phyla, aligning with observations by Zhang and co-workers (2017). In contrast, Chloroflexi showed increased relative abundance in PT and NT, likely due to the absence of GM inputs post-peanut harvest. Actinobacteria, Acidobacteria, and Firmicutes are copiotrophic, thriving on decomposing organic matter with rich nutrients (Clocchiatti et al., 2020), and thus were more abundant in PTGM soils with higher mineral nutrients and SOM. Actinobacteria, known for producing antibiotics like streptomycin and tetracycline, contribute to soil health and are advantageous in farming soils (Marta et al., 2014). Acidobacteria, specialized in degrading SOM, also increased proportionally in PTGM treated (Naumoff and Dedysh, 2012).

The most prevalent fungi in the soil belonged to the phylum Ascomycota, followed by Mortierellomycota, unclassified fungi, and Basidiomycota, consistent with recent global surveys of soil fungal species (Tedersoo et al., 2014). However, our study noted a lesser dominance of Basidiomycota compared to some previous findings, which could be attributed to differences in the duration of continuous cropping (Maestre et al., 2015). Basidiomycota members are known for their involvement in the degradation of biopolymers and their roles in carbon and nitrogen cycling (Justin et al., 2012), and their abundance is affected by soil management practices (Buckley et al., 2006). Basidiomycota also contributes to plant adaptation and stress resistance by enhancing water and nutrient absorption and mitigating the effects of drought, salt, and heavy metals (Hagenbo et al., 2018). Although no obvious difference in Ascomycota abundance was found between PTGM and PT, GM application increased Basidiomycota abundance relative to PT and NT. These findings suggest that green manure application may suppress fungal pathogens and promote beneficial fungal genera. Ding et al. (2021) demonstrated that green manure facilitates the enhancement of soil fungal community structure for pathogen inhibition and nutrient uptake. The shifts in soil microbial community compositions across different tillage practices highlight the potential of green manure application to promote healthy soil ecosystems.

### 4.4 Relationships between microbial community compositions and soil chemical characteristics

The association between microbial community compositions and soil chemical characteristics is pivotal, explaining 86.42% of the variance in our study (Figure 4A). It is evident that shifts in bacterial distribution are related to pH, NH+4-N, NO− 3-N, AP, and SOM contents, consistent with previous findings emphasizing nitrogen and phosphorus as vital nutrients for bacterial proliferation (Gong et al., 2009; Yang et al., 2023). In addition, GM applications could regulate soil microbial community composition by C/N, it has been revealed that higher C/N values could result in a competitive advantage for soil microbial (Yang et al., 2019; Mirza et al., 2014), mainly because the supply or ratio of nitrogen, phosphorus and carbon can regulate nutrient diversity and richness, and the microbial community is very sensitive to soil nitrogen and phosphorus nutrient status (Zhang et al., 2017). Moreover, plow tillage reduces soil BD, and improves soil structure and aeration by loosening the soil and alleviating compaction, thereby promoting microbial growth and enriching bacterial diversity (He et al., 2019).

Similarly, soil chemical properties and nutrients account for 92.89% of the variance in soil fungal community structure. The fungal community correlates closely with pH, SOM, AP, and AK, although TN shows weaker associations. Predominant microflora in PTGM is notably influenced by pH, SOM, NO- 3-N, AP, and AK, as these beneficial microbes (such as Actinobacteria, Proteobacteria, Acidobacteria, and Gemmatimonas) thrive in soils rich in SOM and nutrients, stimulated by the incorporation of crop residues (Ghosh et al., 2016). Gao (2009) demonstrated that an increase in SOM enhanced the abundance of beneficial microflora in tomato and cucumber soils, which could generate antibiotics inhibiting fungal pathogens and mitigating the constraints of continuous cropping. Soil pH exhibits a positive correlation with microbial community composition, particularly among fungi in PTGM (Figure 5B), underscoring the profound influence of soil pH changes induced by GM application on fungal communities. Wang et al. (2022) similarly highlighted soil pH as a critical factor shaping fungal community compositions. These responses of microbial communities to shifts in soil chemical characteristics underscore the importance of high SOM and adequate nutrient availability for maintaining soil health. Additionally, other studies suggest that changes in microbial taxa are influenced by the secretion of root compounds and the deposition of self-inhibitory substances in environments with repeated planting (Huang et al., 2000; Wang et al., 2022).

## 5 Conclusion

Plow tillage combined with 8 years of GM application (PTGM) significantly enhances soil physicochemical characteristics, increases soil nutrient content, microbial abundance, and peanut yield compared to PT and NT. Our findings also demonstrate that PTGM enriches microbial diversity and richness, as well as alters microbial community structure and composition. Changes in soil pH, SOM, and mineral nutrients markedly influence microbial community dynamics across diverse tillage practices. Future research will investigate the relationship between microbial diversity and function in prolonged peanut monocropping, and evaluate the potential of long-term GM application to partially replace chemical nitrogen inputs, thereby reducing environmental stress associated with excessive chemical fertilizer use.

## Data Availability

The original contributions presented in the study are included in the article/Supplementary material, further inquiries can be directed to the corresponding authors.

## Footnotes

^1^ https://www.cloud.majorbio.com
