# Supplementary figures and images for: Long-term adoption of plow tillage and green manure improves soil physicochemical properties and optimizes microbial communities under a continuous peanut monoculture system
Source: Front Microbiol. 2025 Jan 9;15:1513528. doi: 10.3389/fmicb.2024.1513528 (PMC11754393; doi:10.3389/fmicb.2024.1513528)

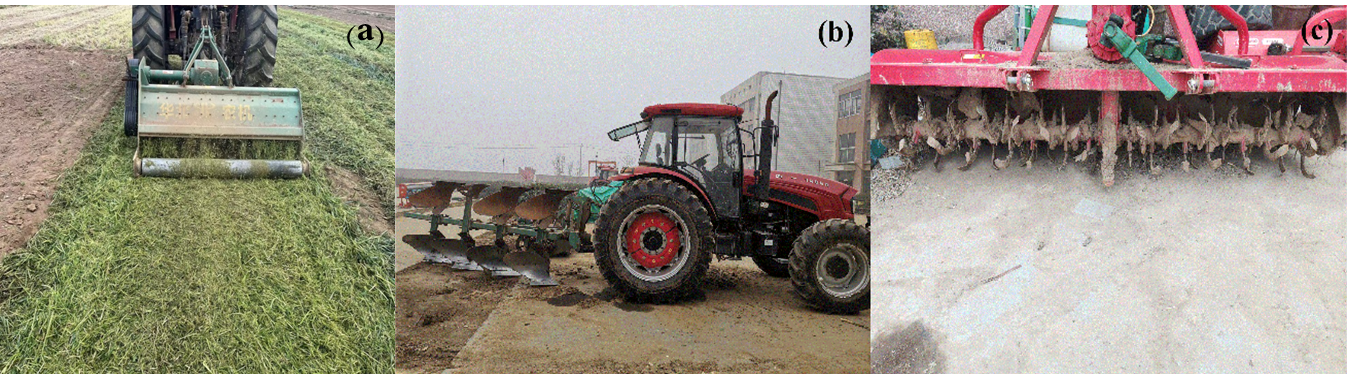

Supplement: Supplementary file 1 [file Image_1.PNG]
